# Interleukin-34 sustains pro-tumorigenic signals in colon cancer tissue

**DOI:** 10.18632/oncotarget.23289

**Published:** 2017-12-15

**Authors:** Eleonora Franzè, Vicenzo Dinallo, Angela Rizzo, Martina Di Giovangiulio, Gerolamo Bevivino, Carmine Stolfi, Flavio Caprioli, Alfredo Colantoni, Angela Ortenzi, Antonio Di Grazia, Giuseppe Sica, Pier Paolo Sileri, Piero Rossi, Giovanni Monteleone

**Affiliations:** ^1^ Department of Systems Medicine, University of Rome “TOR VERGATA”, Rome, Italy; ^2^ Department of Pathophysiology and Transplantation, University of Milan, Milan, Italy; ^3^ Department of Surgery, University “TOR VERGATA” of Rome, Rome, Italy

**Keywords:** IL-34, colorectal cancer, tumorigenesis, M-CSF-1, ERK 1/2

## Abstract

Interleukin-34 (IL-34), a cytokine produced by a wide range of cells, binds to the macrophage colony-stimulating factor receptor (M-CSFR-1) and receptor-type protein-tyrosine phosphatase zeta (PTP-z) and controls myeloid cell differentiation, proliferation and survival. various types of cancers over-express IL-34 but the role of the cytokine in colorectal cancer (CRC) remains unknown. We here investigated the expression and functional role of IL-34 in CRC. A more pronounced expression of IL-34 was seen in CRC samples as compared to matched normal/benign colonic samples and this occurred at both RNA and protein level. Immunohistochemical analysis of CRC tissue samples showed that both cancer cells and lamina propria mononuclear cells over-expressed IL-34. Additionally, CRC cells expressed both M-CSFR-1 and PTP-z, thus suggesting that CRC cells can be responsive to IL-34. Indeed, stimulation of DLD-1 cancer cells with IL-34, but not with MSCF1, enhanced the cell proliferation and cell invasion without affecting cell survival. Analysis of intracellular signals underlying the mitogenic effect of IL-34 revealed that the cytokine enhanced activation of ERK1/2 and pharmacologic inhibition of ERK1/2 abrogated IL-34-driven cell proliferation. Consistently, IL-34 knockdown in HT-29 cells with a specific IL-34 antisense oligonucleotide reduced ERK1/2 activation, cell proliferation and enhanced the susceptibility of cells to Oxaliplatin-induced death. This is the first study showing up-regulation of IL-34 in CRC and suggesting a role for this cytokine in colon tumorigenesis.

## INTRODUCTION

Colorectal carcinoma (CRC) is one of the most common forms of malignancy and the second leading cause of cancer-related death in the Western world [1]. Chronic inflammation is an independent risk factor for the development of CRC [2]. Approximately 2% of CRC cases arise in patients with long-standing and extensive ulcerative colitis, one of the 2 major forms of inflammatory bowel diseases (IBD) in humans, while the majority of CRCs develops in individuals who are not affected by IBD [3]. However, even in these latter patients, the neoplastic tissue is massively infiltrated with immune cells, including T cells, natural killer (NK) cells and macrophages cells, which can either foster or inhibit the survival and growth of CRC cells, mostly through the production of cytokines [4]. Additional protumorigenic signals originate from other cells present in the tumor tissue, such as tumor-associated macrophages and cancer-associated fibroblasts, whose functions are under the control of factors secreted by neoplastic cells, immune cells and stromal cells [5].

Interleukin-34 (IL-34) is a cytokine produced by a wide range of cells, including macrophages, endothelial cells, fibroblasts, neurons and epithelial cells and is constitutively expressed in adult human tissues, such as heart, brain, liver, spleen, thymus, testis, ovary, prostate, small intestine and colon [6–9]. IL-34 shares no apparent sequence homology with macrophage co type lony-stimulating factor (M-CSF-1; also known as CSF-1) but its biological activity is mediated by interaction with the homodimeric M-CSF receptor 1 (M-CSFR-1; also known as CFS-1R or FMS) [10, 11]. M-CSFR-1 is mainly expressed on cell surface of macrophages and to a lesser extent by non-immune cells, such as trophoblasts, osteoclasts, smooth muscle cells and neurons [12–14]. IL-34/M-CSFR-1 signaling regulates myeloid cell differentiation, proliferation and survival and enhances secretion of pro-inflammatory cytokines and chemokines in various systems [12, 15–18]. A second uncovered receptor of IL-34, PTP-ζ, has been recently described [19].

Elevated levels of IL-34 are detectable in patients with various types of cancers, such as blood, brain, breast, eye, head and neck, lung, ovarian and skin cancer, and it has been demonstrated that IL-34 expression is associated with the progression of such tumours [6, 20–24]. There is also evidence that IL-34 plays a role in tumorigenesis given its ability to stimulate endothelial cell proliferation, vascular cord formation and recruitment of macrophages into the tumor tissue [20, 25]. However, the role of IL-34 in sporadic CRC is unknown. We here investigated the expression and functional role of IL-34 in CRC.

## RESULTS

### Sporadic colorectal cancer over-expresses IL-34

**Figure 1:**
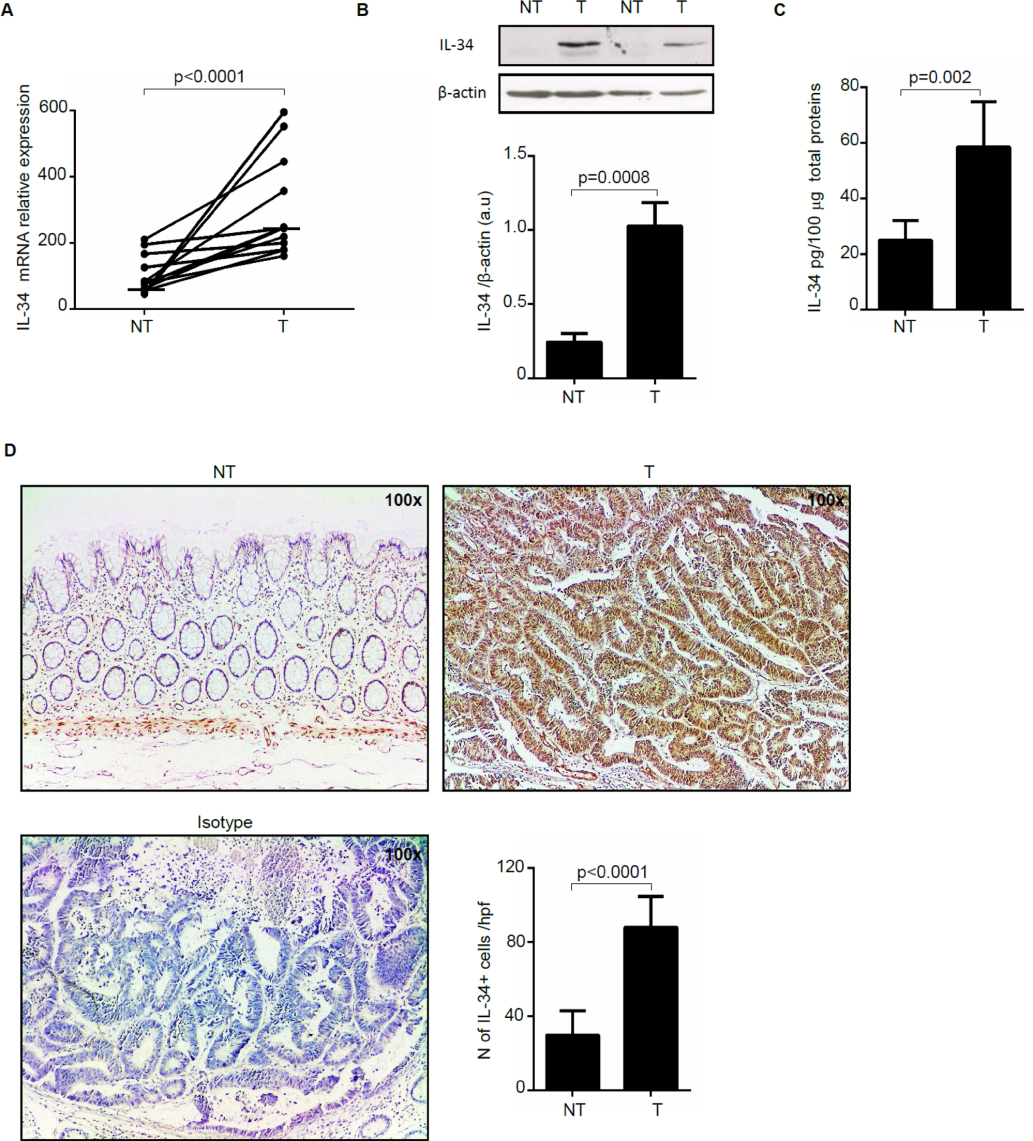
Interleukin-34 (IL-34) RNA transcripts and protein are increased in sporadic colorectal cancer (CRC) (**A**) Paired colonic explants taken from non-tumoral (NT) and tumoral (T) areas of 12 patients with sporadic CRC were analyzed for IL-34 RNA expression by real-time PCR and levels were normalized to β-actin. Each point represents the value of IL-34 mRNA in a single patient and horizontal bars indicate the median value. (**B**) Representative Western blots showing IL-34 and β-actin in total proteins extracted from paired non-tumoral (NT) and tumoral (T) areas of two patients with sporadic CRC. Bottom panels show the quantitative analysis of IL-34/β-actin ratio in samples taken from paired non-tumoral (NT) and tumoral (T) areas of 5 CRC patients as measured by densitometry scanning of Western blots. Values are expressed in arbitrary units (a.u.) and indicate mean ± SEM of all samples. (**C**) Quantitative analysis of IL-34 protein expression in paired colonic explants taken from the non-tumoral (NT) and tumoral (T) areas of 8 patients with sporadic CRC were measured by ELISA. Data were expressed as mean ± SEM of all samples. (**D**) Representative photomicrographs (100× original magnification) of IL-34-stained frozen sections of surgical specimens taken from non-tumoral (NT) and tumoral (T) areas of one patient with sporadic CRC. Staining with isotype control antibody is also shown. Right panel shows the number of IL-34-positive cells for high power field (hpf) of 8 separate experiments in which sections of 8 CRC patients were analyzed. Data are expressed as mean ± SEM.

To examine if IL-34 expression is differently regulated during CRC, total RNA was extracted from paired colonic samples of non-tumoral and tumoral areas of patients with sporadic CRC and analyzed by real-time PCR. Tumoral samples expressed higher IL-34 RNA levels than non-tumoral samples of the same CRC patients (Figure 1A). Western blotting of total proteins extracted from freshly obtained samples and densitometry analysis of blots confirmed up-regulation of IL-34 in the tumoral samples (Figure 1B). Consistently, IL-34 protein level measured by ELISA was significantly increased in extracts of tumoral area than in those of non-tumoral area of CRC patients (Figure 1C). Immunohistochemistry analysis of CRC sections showed that IL-34 was mostly produced by cancer cells and to lesser extent by lamina propria mononuclear cells (LPMC) while staining of normal colonic sections revealed that IL-34 was weakly expressed by LPMC and virtually absent in epithelial cells (Figure 1D).

To examine whether IL-34 production is regulated by inflammatory cytokines that are over-produced in CRC tissue [26–29], DLD-1 cells were stimulated with TNF-α, IL-6, IL-17A and IFN-γ and IL-34 secretion was evaluated by ELISA. In each of 3 independent experiments, all these cytokines enhanced IL-34 secretion (Table 1).

**Table 1:** IL-34 is induced by TNF-α, IL-6, IL-17A and IFN-γ in DLD-1 cancer cells

Experiment	UNST	TNF-α (20 ng/ml)	IL-6 (50 ng/ml)	IL-17A (20 ng/ml)	IFN-γ (100 ng/ml)
1°	44,6	114,8	81,9	82,8	119
2°	27,1	53,9	54,9	57	143
3°	28,1	51,9	40,5	54,9	155

DLD-1 cells were stimulated with TNF-α (20 ng/ml), IL-6 (50 ng/ml), IL-17 A (20 ng/ml) and IFN-γ (100 ng/ml). After 48 hours, cell-free culture supernatants were collected and analyzed by ELISA. Data are expressed as pg/ml and indicate the values of IL-34 measured in supernatants of 3 independent experiments

### Enhanced expression of M-CSFR-1 in CRC

**Figure 2:**
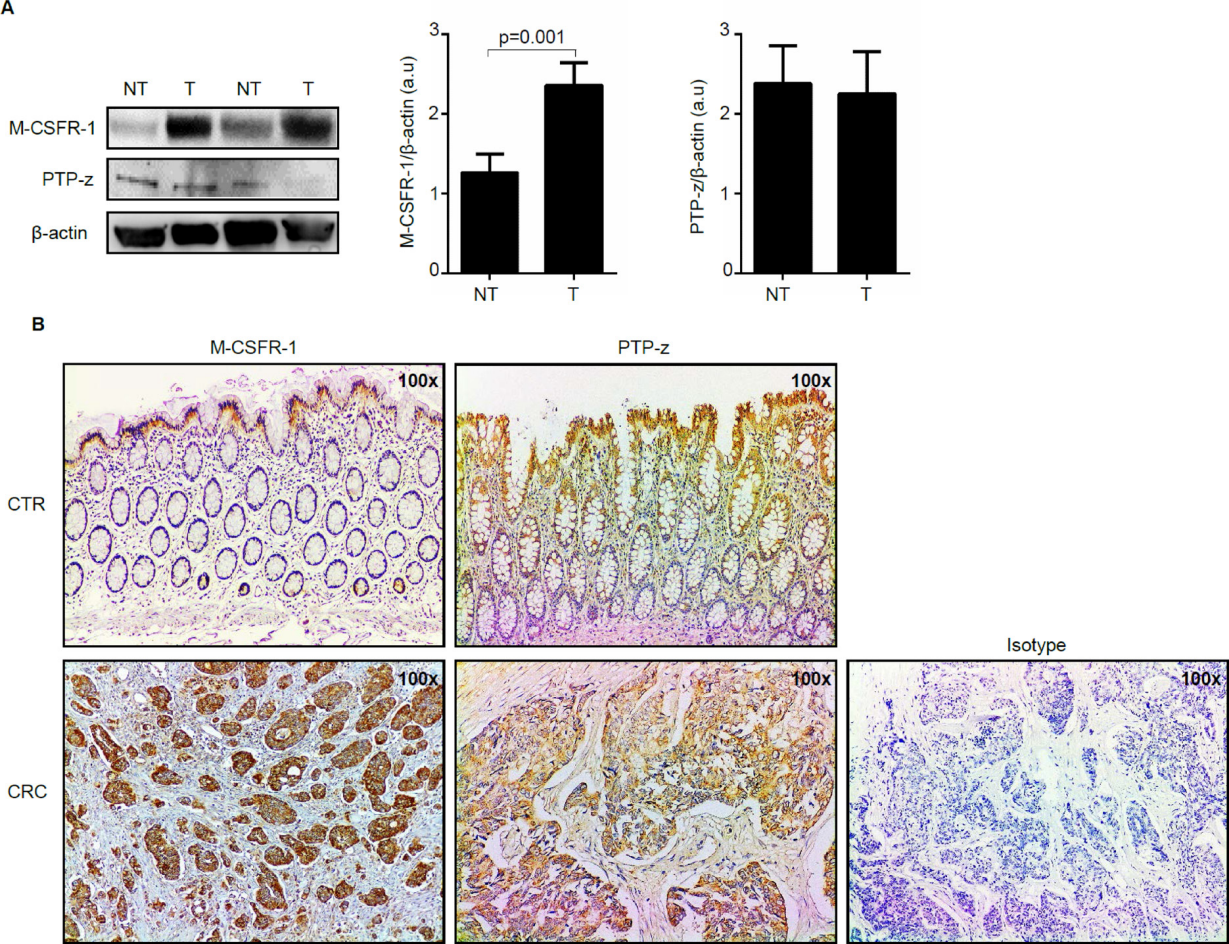
Macrophage colony-stimulating factor -1 receptor (M-CSFR-1) but not Receptor-type tyrosine-protein phosphatase zeta (PTP-z) is increased in sporadic colorectal cancer (CRC) (**A**) Representative Western blots showing M-CSFR-1, PTP-z and β-actin in total proteins extracted from paired non-tumoral (NT) and tumoral (T) areas of 2 patients with sporadic CRC. Right panels show the quantitative analysis of M-CSFR-1/β-actin and PTP-z/β-actin ratio respectively in paired non-tumoral (NT) and tumoral (T) areas taken from 5 CRC patients as measured by densitometry scanning of Western blots. Values are expressed in arbitrary units (a.u.) and indicate mean ± SEM of all samples. (**B**) Representative photomicrographs (100x original magnification) of M-CSFR-1-and PTP-z stained paraffin-embedded sections of surgical colonic samples taken from one control (CTR) and one patient with sporadic CRC. The figure is representative of 3 separate experiments in which sections taken from 4 CTR and 3 patients with CRC were analyzed for M-CSFR-1 and PTP-z. Staining with isotype control antibody is also shown.

To begin to examine whether IL-34 controls CRC cell behavior, we analyzed expression of IL-34 receptors in paired colonic samples taken from tumoral and non-tumoral areas of CRC patients. M-CSFR-1 expression quantitated by densitometry and normalized by β-actin expression was significantly increased in proteins extracted from tumoral areas as compared to samples of non-tumoral areas (Figure 2A). Immunohistochemical analysis confirmed such data and showed that CRC cells were strongly positive for this receptor (Figure 2B). Western blotting showed that PTP-z was detectable in all the samples with no apparent difference between tumoral and non-tumoral areas (Figure 2A). Immunohistochemical analysis showed that such a receptor was expressed by both epithelial cells and LPMC in non-tumoral and tumoral areas (Figure 2B). Consistently, both receptors were seen in HCT-116 and DLD-1, two CRC cell lines (Supplementary Figure 1). Altogether, these data indicate that CRC cells have the potential to functionally respond to locally-produced IL-34.

### IL-34 enhances CRC cell proliferation and cell invasion

**Figure 3:**
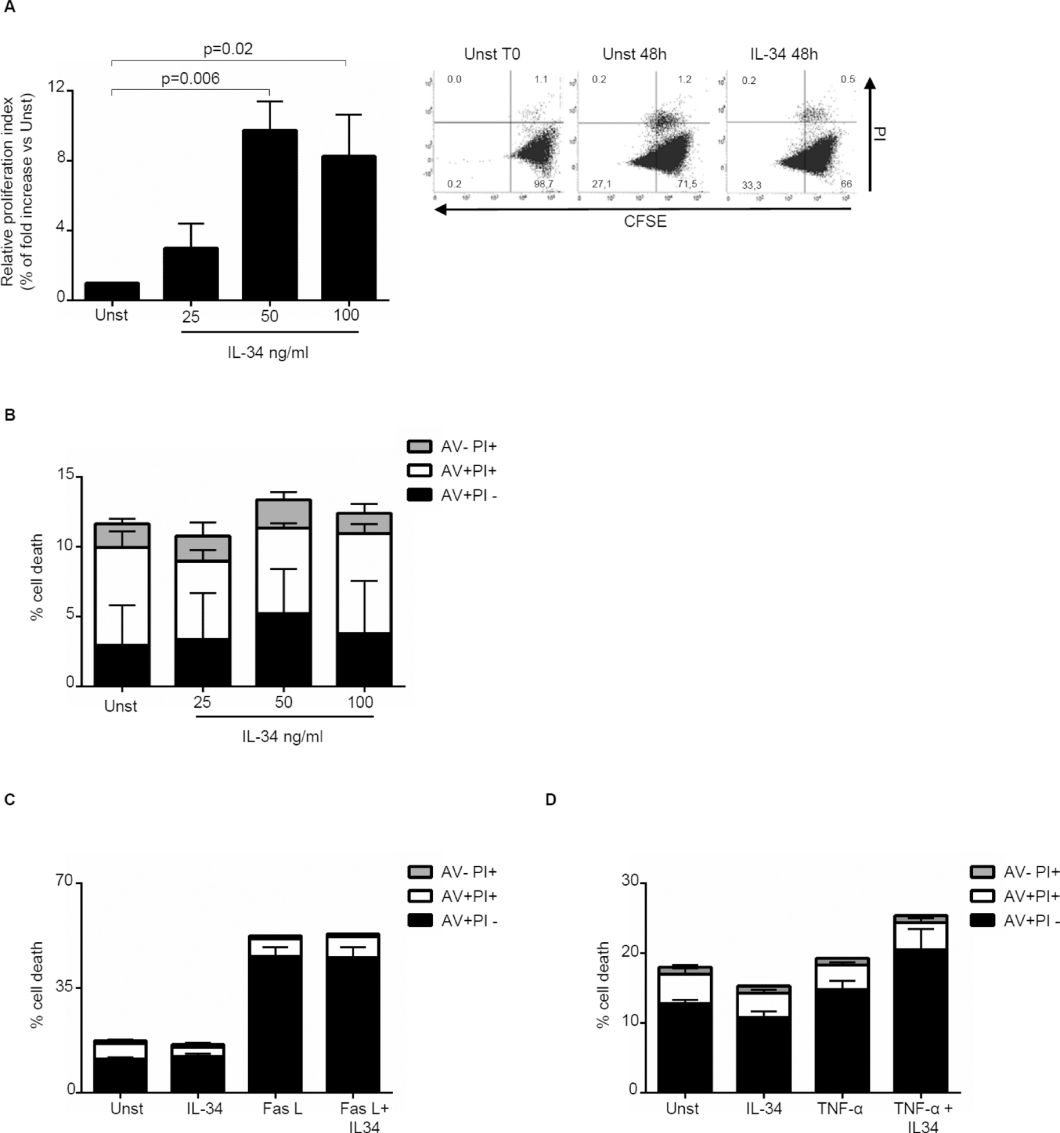
Interleukin-34 (IL-34) induces CRC cell proliferation but does not affect cell death (**A**) Serum-starved DLD-1 cells were either left unstimulated (Unst) or stimulated with increasing doses of recombinant human IL-34 (25–100 ng/ml) for 48 hours. Cell proliferation was evaluated by flow cytometry and proliferation index was calculated with Modfit LT. Data indicate mean ± SEM of 4 independent experiments. Right insets: representative dot-plots showing the percentages of CFSE- and/or PI-positive cells before and after IL-34 stimulation. (**B**) Serum-starved DLD-1 cells were either left unstimulated (Unst) or stimulated with increasing doses of recombinant human IL-34 (25–100 ng/ml) for 48 hours. Data indicate the percentage of cell death as assessed by flow cytometry analysis of AV and/or PI-positive cells and are expressed as mean ± SEM of 4 experiments. (**C**) Serum-starved DLD-1 cells line were either left unstimulated (Unst) or stimulated with recombinant human IL-34 (50 ng/ml) and/or Fas Ligand (Fas L) (200 ng/ml) for 48 hours. The percentage of cell death was assessed by flow cytometry analysis of AV and/or PI-positive cells. Data indicate mean ± SEM of 4 independent experiments. (**D**) Serum-starved DLD-1 cells were either left unstimulated (Unst) or stimulated with recombinant human IL-34 (50 ng/ml) and/or TNF-α (100 ng/ml) for 48 hours. The percentage of cell death was assessed by flow cytometry analysis of AV and/or PI-positive cells. Data indicate mean ± SEM of 4 independent experiments.

**Figure 4:**
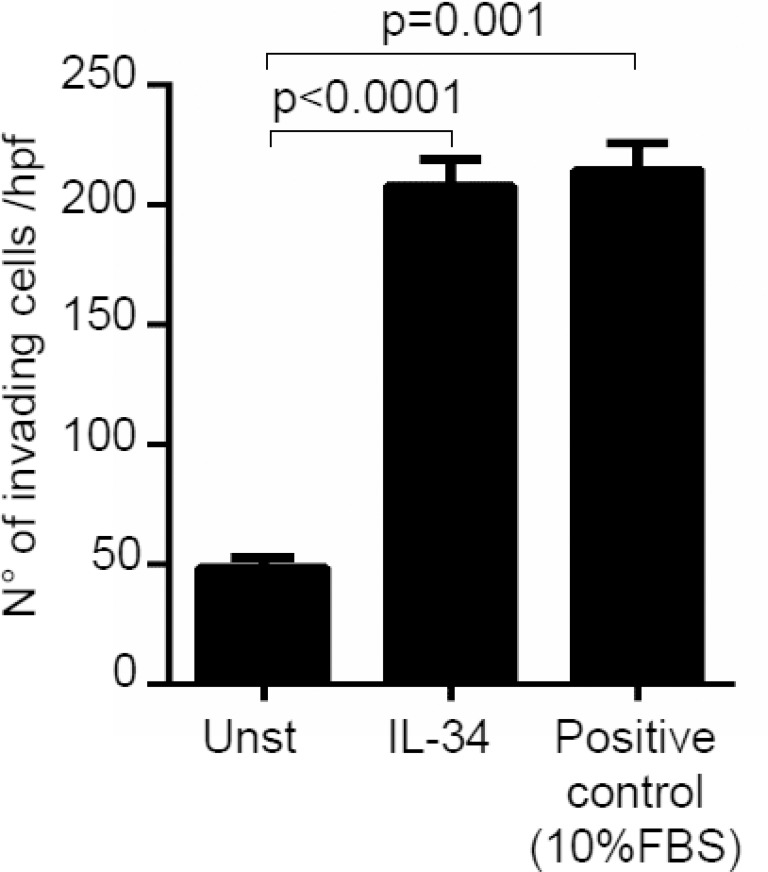
Interleukin-34 (IL-34) induces CRC cell invasion DLD-1 cells (3 × 10^4^) were seeded in Transwell inserts precoated with Matrigel, and either left unstimulated (Unst) or stimulated with IL-34 (50 ng/ml) or 10% fetal bovine serum (FBS) (used as positive control of invasion) for 48 hours. Data are expressed as mean ± SEM of three independent experiments.

**Figure 5:**
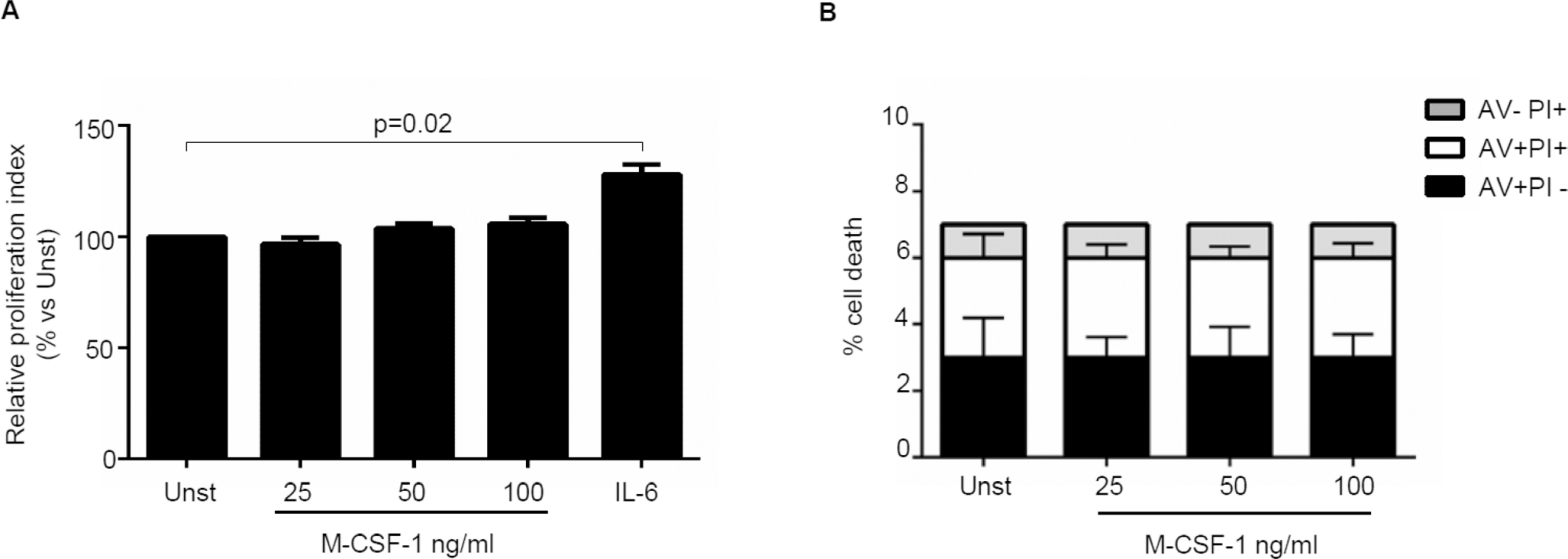
Macrophage colony-stimulating factor -1 (M-CSF-1) does not affect CRC cell growth and death (**A**) Serum-starved DLD-1 cells were either left unstimulated (Unst) or stimulated with increasing doses of recombinant human M-CSF-1 (25–100 ng/ml) or IL-6 (50 ng/ml, used as a positive control) for 48 hours. Cell proliferation was evaluated with CFSE staining by flow cytometry. Data indicate mean ± SEM of four independent experiments. (**B**) Serum-starved DLD-1 cells were either left unstimulated (Unst) or stimulated with increasing doses of recombinant human M-CSF-1 (25–100 ng/ml) for 48 hours. The percentage of cell death was assessed by flow cytometry analysis and expressed as percentage of AV and/or PI-positive cells. Data are expressed as mean ± SEM of 6 independent experiments.

Next, we investigated whether IL-34 controls CRC cell growth, survival and invasion. IL-34 enhanced DLD-1 and HT-29 cell growth, an effect that was mainly evident at a final concentration of 50 ng/ml (Figure 3A, Supplementary Figure 2A). In contrast, no significant change in the percentages of Annexin V (AV)/propidium iodide (PI)-positive cells was seen following stimulation of CRC cells with IL-34 (Figure 3B, Supplementary Figure 2B). Similarly, IL-34 did not significantly affect the fractions of AV/PI-positive cells induced by FAS Ligand or TNF-α (Figure 3C–3D). The proliferative effect of IL-34 appeared to be specific on CRC cells, as IL-6 but not IL-34 increased the growth of HCEC-1C, a normal colon epithelial cell line (Supplementary Figure 2C). Moreover, by using a matrigel invasion assay, we showed that IL-34 enhanced DLD-1 cell invasion (Figure 4). No significant change in cell proliferation and viability was seen following stimulation of DLD-1 cells with M-CSF-1 (Figure 5A–5B).

### IL-34 stimulates CRC cell proliferation via ERK1/2-dependent pathway

**Figure 6:**
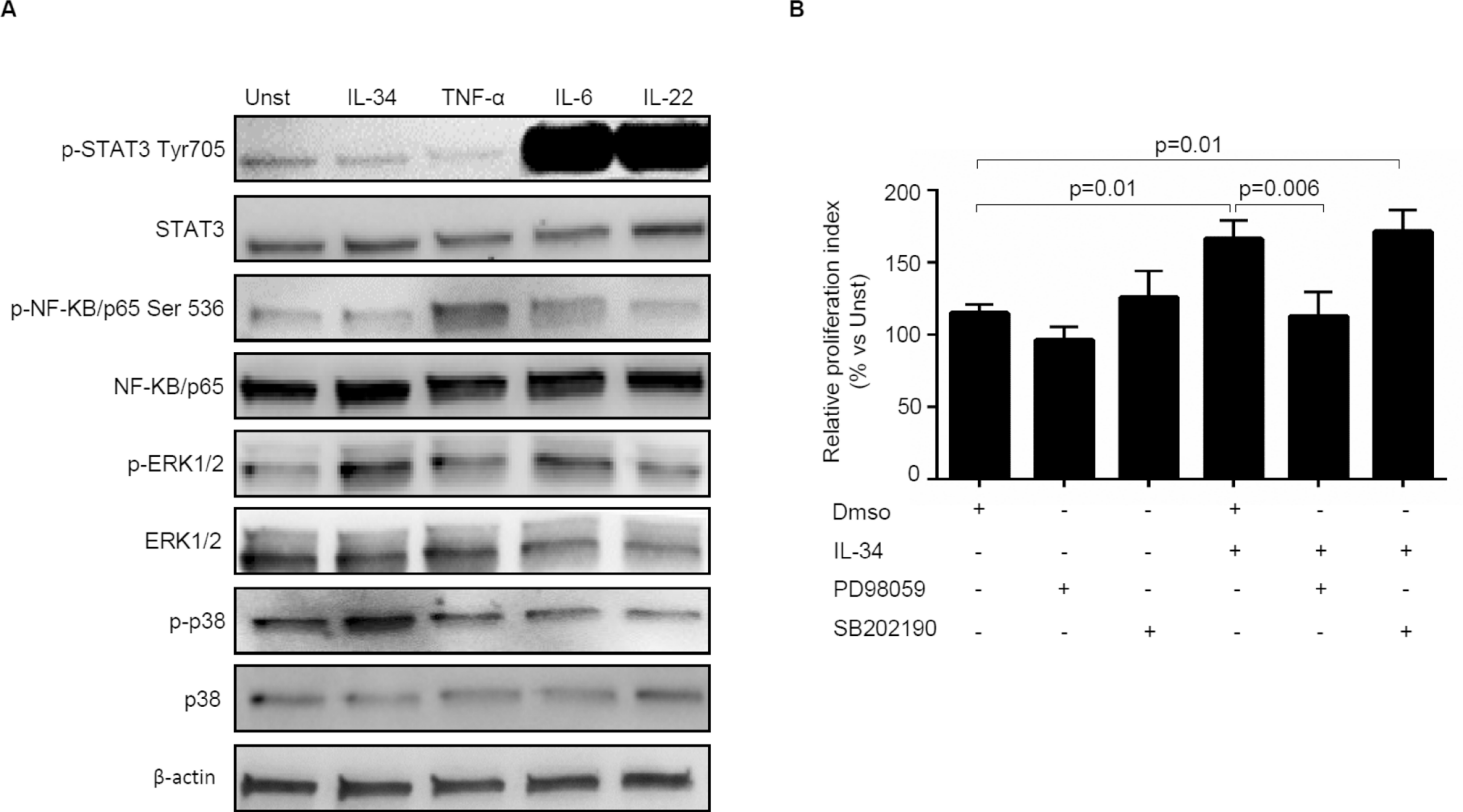
Interleukin-34 (IL-34) induces CRC cell proliferation through an ERK1/2-dependent mechanism (**A**) Serum-starved DLD-1 cells were either left unstimulated (Unst) or stimulated with IL-34 (50 ng/ml), TNF-α (25 ng/ml), IL-6 (25 ng/ml), IL-22 (25 ng/ml) for 15 minutes. Phosphorylated and total forms of the STAT3, NF-KB/p65 , ERK1/2, p38, and β-actin were evaluated by Western blotting. One of 4 independent experiments is shown. (**B**) Serum-starved DLD-1 cells were pre-incubated with either specific inhibitors of ERK1/2 and p38 (PD98059 and SB202190 respectively) or with Dimethyl sulfoxide (DMSO, vehicle) for 1 hour and then stimulated with IL-34 (50 ng/mL) for further 48 hours. Cell proliferation was evaluated by flow cytometry. Data indicate mean ± SEM of 4 independent experiments.

In the subsequent studies, we explored the basic mechanism by which IL-34 regulates positively CRC cell growth. Initially, we examined whether IL-34 activates signalling pathways that control neoplastic cell proliferation. To this end, DLD-1 cells were either left unstimulated or stimulated with IL-34 or TNF-α, IL-6 or IL-22, which were used as positive inducers of MAP Kinases, NF-Kβ and STAT3 signalling respectively [30–34]. IL-34 enhanced phosphorylation of both ERK1/2 and p38 MAP Kinases without affecting phosphorylation of STAT3 and NF-Kβ /p65 (Figure 6A). To test whether ERK1/2 and p38 activation mediates IL-34-driven DLD-1 cell growth, cells were pre-incubated with specific inhibitors of these two MAP Kinases prior to being stimulated with IL-34. Treatment of DLD-1 cells with PD98059, an inhibitor of ERK1/2, but not with SB202190, a p38 inhibitor, abrogated IL-34-induced DLD-1 cell proliferation (Figure 6B).

### IL-34 knockdown with a specific antisense oligonucleotide reduces CRC cell growth

**Figure 7:**
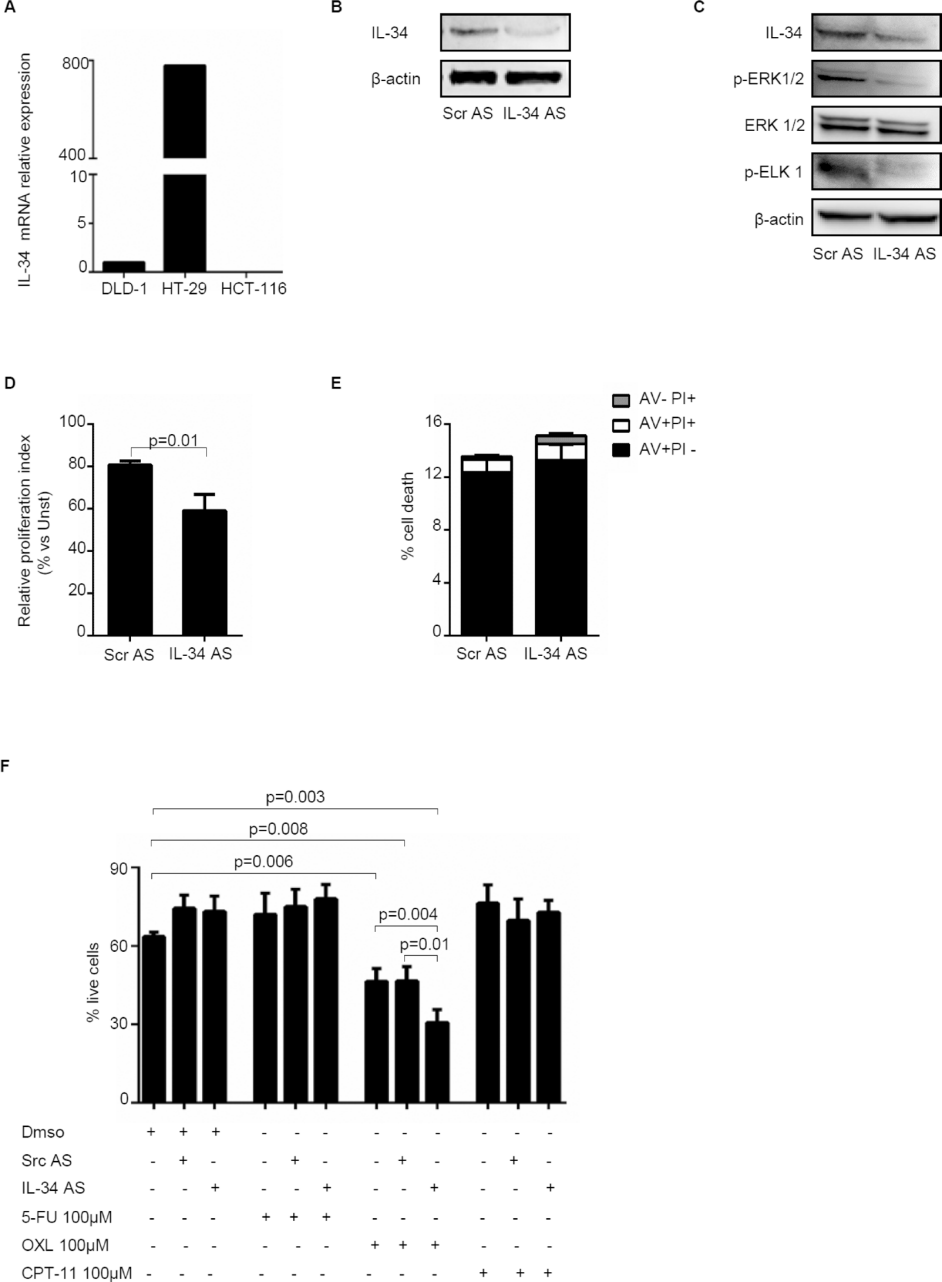
Knockdown of IL-34 with a specific antisense oligonucleotide decreases HT-29 cell proliferation and increases oxaliplatin-induced cell death (**A**) IL-34 was evaluated in the human CRC cell lines DLD-1, HT-29 and HCT-116 by real-time PCR and levels were normalized to β-actin. (**B**) HT-29 cells were transfected with scrambled negative control oligonucleotide (SCR AS) or IL-34 antisense oligonucleotide (IL-34 AS) (both used at 2 µg/ml) for 48 hours. IL-34 and β-actin were analyzed by Western blotting. One of 4 independent experiments is shown. (**C**) HT-29 cells were transfected with either SCR AS or IL-34 AS (both used at 2 µg/ml) for 72 hours. Phosphorylated and total forms of ERK1/2, ELK 1, IL-34 and β-actin were evaluated by Western blotting. One of 4 independent experiments is shown. (**D**) HT-29 cells were transfected with either SCR AS or IL-34 AS (both used at 2 µg/ml) for 72 hours. Cell proliferation was evaluated by flow cytometry following staining with CFSE. Data indicate mean ± SEM of 4 independent experiments. (**E**) HT-29 cells were transfected with either SCR AS or IL-34 AS (both used at 2 µg/ml) for 72 hours. The percentage of cell death was assessed by flow cytometry. Data are indicated as percentage of AV and/or PI-positive cells and expressed as mean ± SEM of 4 indipendent experiments. (**F**) HT-29 cells were transfected with either SCR AS or IL-34 AS (both used at 2 µg/ml) for 24 hours and then stimulated with 5-Fluorouracile (5-FU), Oxaliplatin (OXL) and Irinotecan (CPT-11) (all used 100 µM) or DMSO (vehicle) for further 24 hours. Data are expressed as mean ± SEM of 4 independent experiments.

To confirm that CRC cell-derived IL-34 regulates positively CRC cell proliferation, we inhibited IL-34 in HT-29 cells with a specific IL-34 antisense oligonucleotide. For these experiments, we selected HT-29 cells as this cell line expresses elevated levels of IL-34 (Figure 7A). Treatment of HT-29 cells with the specific IL-34 antisense oligonucleotide decreased IL-34 protein expression (Figure 7B) and phosphorylation of ERK1/2 and ELK1, a down-stream target of ERK1/2 (Figure 7C). IL-34 knockdown was associated with a significant reduction of cell growth (Figure 7D) but no change in cell viability (Figure 7E). Finally, treatment of HT-29 cells with IL-34 antisense oligonucleotide enhanced the susceptibility of cells to oxaliplatin (OXL)-induced death while cells were resistant to 5-Fluorouracile (5-FU) and Irinotecan (CPT-11), regardless of whether IL-34 expression was silenced (Figure 7F).

## DISCUSSION

Although the exact sequence of events that sustain CRC growth remains poorly characterized, there is evidence that cytokines produced by immune cells and stromal cells exert proliferative effects on CRC cells [4, 35]. On the other hand, CRC cells can produce additional factors that acting in a paracrine/autocrine manner expand further cell growth [36]. Identifying such molecules and the molecular mechanisms underlying CRC cell growth could help design novel therapeutic strategies for patients with advanced CRC.

The present study was undertaken to examine the expression and functional role of IL-34 in human CRC. By using various assays, we initially showed that IL-34 is constitutively expressed in the normal colon and its expression is increased in tumoral samples of CRC patients. Analysis of cell sources of IL-34 showed that both cancer cells and mononuclear cells infiltrating the tumoral tissue produce elevated levels of the cytokine. Normal colonic mucosa, adjacent to the tumoral area, expressed low levels of IL-34, thus suggesting that stimuli generated within the tumoral microenvironment can elicit IL-34 production. In this context, data of the present study indicate that cytokines over-produced by immune cells infiltrating the CRC tissue, such as TNF-α, IL-6, IL-17A and IFN-γ [26–29], enhance IL-34 production. The fact that IL-34 expression was enhanced by IFN-γ could appear inconsistent with the notion that IFN-γ stimulates anti-tumor adaptive immune response. However, there is evidence that, in specific conditions IFN-γ is pro-tumorigenic [29, 37].

We show also CRC cells express both M-CSFR-1 and PTP-z, the two known IL-34 receptors, and, therefore, are potentially capable of responding to the cytokine. Indeed, stimulation of CRC cells with IL-34 resulted in enhanced cell growth and cell invasion, while the cytokine did not alter CRC cell viability. Treatment of CRC cells with M-CSF-1, another ligand of M-CSFR-1, associated with no change in cell growth and survival. These findings are in line with results of previous studies showing that IL-34 and M-CSF-1 exert non-redundant functions though they share a common receptor [10]. The divergent functions of these two cytokines could rely on their ability to differently bind and signal through the receptor as previous studies showed that IL-34 is more powerful than M-CSF-1 in triggering M-CSFR-1 activation [38]. The fact that CRC cells express both IL-34 and IL-34 receptors raises the possibility that IL-34 acts in an autocrine and/or paracrine manner to regulate CRC cell growth. This hypothesis is supported by the demonstration that knockdown of IL-34 with a specific antisense oligonucleotide in HT-29 cells reduced cell growth without affecting cell apoptosis/necrosis. Moreover, IL-34 knockdown enhanced the susceptibility of CRC cells to OXL-induced death. These results are consistent with studies in lung cancer showing that IL-34, produced during chemotherapy, enhances cancer cell survival [22].

Evaluation of the intracellular pathways sustaining CRC cell growth revealed that IL-34 activates ERK1/2 and p38 MAP kinases. However, pharmacological inhibition of ERK1/2 but not of p38 abrogated the ability of IL-34 to enhance CRC cell growth. These findings are in line with the demonstration that M-CSFR-1 has a tyrosine kinase activity and interaction with IL-34 induces the phosphorylation of a tyrosine residue of the receptor cytoplasmic domain and its homodimerisation, with the downstream effect of initiating a cascade of phosphorylation of other proteins, including ERK1/2 [10–12].

Recent studies showed a positive correlation between IL-34 expression and tumor development. For example, IL-34 promotes osteoclastogenesis and appears to be involved in giant cell tumors of bone [6]. Initial evidence suggests the involvement of IL-34 in mammary cancer in which IL-34 levels have been associated with shorter survival and time to recurrence following cytotoxic therapies [24]. In such a neoplasia, as well as in other tumors (i.e. teratoma, hepatocellular carcinoma), the pro-tumoral effect of IL-34 would be linked to the ability of the cytokine to promote the survival and the differentiation of type 2 macrophages [23, 25, 39, 40]. Data of the present study expand on these observations and indicate that IL-34 can directly target cancer cells and stimulate intracellular pathways that positively regulate colon carcinogenesis. This does not however exclude the possibility that IL-34 can control the function of additional cell types, which in turn sustain CRC cell proliferation. For instance, IL-34 stimulates intestinal immune cells to make TNF-α and IL-6, two cytokines exerting proliferative effects on CRC cells, and has been involved in the suppressive function of regulatory T cells, a subset of T cells whose activity associates with the progression of cancer cells [18, 26, 41–43]. Moreover, IL-34 facilitates polarization of memory T cells into Th17 cells, which enhance cancer cell growth in many organs [44].

In conclusion, this is the first study showing up-regulation of IL-34 in CRC and suggesting a role for this cytokine in the growth of CRC cells.

## METHODS

### Patients and samples

Paired tissue samples were taken from the tumoral area and the macroscopically and microscopically unaffected, adjacent colonic mucosa of 26 patients who underwent colon resection for sporadic CRC at the Tor Vergata University Hospital (Rome, Italy) or Fondazione IRCCS Cà Granda, Ospedale Maggiore Policlinico (Milan, Italy). All patients received neither radiotherapy nor chemotherapy prior to undergoing surgery.

Each patient who took part in the study gave written informed consent and the study protocol was approved by the local Ethics Committee (Tor Vergata University Hospital, Rome. Protocol number:171/16).

### Intestinal epithelial cell lines

The human colon cancer cell lines DLD-1 (phenotype: k-ras mutation G13D, p53 wild type), HT-29 (phenotype: k-ras wild type, mutated p53) and HCT-116 (phenotype: : k-ras mutation G13D, p53 wild type) were cultured in 25 cm^2^ plastic flasks and maintained at 37°C in a humidified atmosphere of 5% CO_2_ in RPMI 1640 or McCoy’s 5A (all from Lonza, Verviers, Belgium) supplemented with 10% fetal bovine serum (FBS), penicillin (P) (100 U/ml), streptomycin (S) (100 μg/ml) (all from Lonza). Human colon epithelial cells (HCEC-1CT) were cultured in 25 cm^2^ plastic flasks and maintained at 37°C in a humidified atmosphere of 5% CO_2_ in medium ColoUpTm (Evercyte GmbH, Muthgasse, Vienna).

To evaluate whether IL-34 or M-CSF-1 regulate cancer cell growth and viability, 2 × 10^5^ DLD-1 cells, HT-29 cells or HCEC-1CT cells were plated into each well of a 6-wells plate and left to adhere. After 24 hours, cells were starved for 12 hours and then stimulated with recombinant human IL-34 or M-CSF-1 (both used at a final concentration of 25–100 ng/mL, R&D Systems, Inc. Minneapolis, MN, USA) or IL-6 (50 ng/ml, R&D Systems) in fresh medium containing 0,05% bovine serum albumin (BSA) (Sigma) for 48 hours. To evaluate if IL-34 synergizes with apoptotic stimuli, serum-starved DLD-1 cells were either left unstimulated or stimulated with recombinant human IL-34 (50 ng/mL) in presence or absence of FasL (200 ng/ml, Enzo Life, Sciences, NY, USA) or TNF-α (100 ng/ml, R&D Systems) for 48 hours. At the end of cell culture, cell death was assessed by flow cytometry.

To determine the factors that modulate IL-34 expression, DLD-1 cells were either left unstimulated or stimulated with TNF-α (20 ng/ml), IL-6 (50 ng/ml), IL-17A (20 ng/ml, R&D Systems) or IFN-γ (100 ng/ml, Peprotech Ec Ltd,). After 48 hours, cell-free supernatants were analyzed by ELISA. To examine the molecular mechanism that controls the proliferative effect of IL-34 on cancer cells, serum-starved DLD-1 cells were either left unstimulated or stimulated with recombinant human IL-34 (50 ng/mL), TNF-α (25 ng/mL), IL-6 (25 ng/mL) and IL-22 (25 ng/ml) for 15 minutes, then lysed and total extracts were analyzed for the content of both phosphorylated and total form of MAP kinases (ERK1/2, p38), NF-kB p65 and Stat3 by Western blotting. To evaluate the effect of MAP Kinase on IL-34-induced cancer cell proliferation, serum-starved DLD-1 cells were preincubated with PD98059 (50 µM, EMD Millipore Corporation, Billerica, MA, USA), a selective inhibitor of ERK1/2, or SB202190 , an inhibitor of p38 (10 mol/L, EMD Millipore) or dimethyl sulfoxide (DMSO; vehicle, Sigma-Aldrich, Milan, Italy) for 1 hour and then stimulated or not with IL-34 (50 ng/ml) for 48 hours. The specificity of both ERK1/2 and p38 inhibitors has been previously verified [18]. At the end of cell culture, cell proliferation was assessed by flow cytometry.

In additional experiments, HT-29 cells were either left untreated or transfected with a specific IL-34 antisense oligonucleotide or scrambled antisense oligonucleotide (both used a 2 µg/ml, Exiqon, Woburn, MA, USA) for 48–72 hours using Opti-MEM medium and Lipofectamine 3000 reagent (both from Life Technologies, Milan, Italy) according to the manufacturer’s instructions. At the end, cell viability and proliferation and ERK1/2 activation were evaluated. The efficiency of the transfection was determined by Western blotting. To evaluate the effect of IL-34 on chemotherapeutic agent-induced CRC cell death, HT-29 cells were transfected with the specific IL-34 antisense oligonucleotide or scrambled antisense oligonucleotide (both used a 2 µg/ml, Exiqon,) for 24 hours and then stimulated with 5-FU, OXL or CPT-11 (all used 100 µM, Sigma) or DMSO (vehicle) for further 24 hours. At the end, cell viability was evaluated by flow cytometry.

### Analysis and quantification of cell proliferation and death

Cell proliferation was assessed by flow cytometry using carboxyfluorescein diacetate succinimidyl ester (CFSE; Molecular Probes, Life Technologies), which covalently binds cell components to yield a fluorescence that is divided equally between daughter cells at each division.

Briefly, starved DLD-1 or HT-29 cells were incubated with CFSE according to the manufacturer’s instructions. After 30 min, the medium was removed and fresh medium was added for the indicated time points. At the end, cells were collected, washed twice with PBS, and then incubated with PI (5 mg/ml, Life Technologies) for 15 min at 4°C in the dark. CFSE- and/or PI-positive cells were determined by flow cytometry (FACSVerse BD Biosciences, San Jose, CA, USA) and the data were analyzed using ModFit LT 5.0 (Verity Software House, Inc., Topsham, ME, USA) and expressed as proliferation index relative to unstimulated conditions.

To score cell death, DLD-1 or HT-29 cells, left untreated or stimulated as described above for the indicated time points, were washed in PBS, stained with FITC-AV (1:100 final dilution, Immunotools, Friesoyte, Germany) according to the manufacturer’s instructions and incubated with 5 mg/ml PI (Life Technologies) for 20 min at 4°C. Then, the fluorescence was measured by flow cytometry using FL-1 and FL-2 channels of FACSVerse (BD Biosciences) flow cytometer. Viable cells were considered as AV-/PI-cells, apoptotic cells as AV+/PI-cells, whereas secondary necrotic cells were characterized by AV+/PI+ staining. Data are expressed as percentage of cell death.

### Cell invasion assay

To evaluate if IL-34 promotes CRC cell invasion, transwell inserts (Corning, Inc., Corning, NY, USA) were precoated with Matrigel matrix (BD Biosciences, Franklin Lakes, NJ, USA) mixed 1:1 with RMPI 1640 (Lonza) at 37°C for 6 hours. DLD-1 cells were trypsinized and plated into the upper chambers at a concentration of 3 × 10^4^ cells/100 µl in serum-free RPMI 1640 with 0,05% of BSA (Sigma). The lower chambers were filled with 800 µL RPMI-1640 medium containing 0,05% BSA and IL-34 (50 ng/ml) or 10% FBS (Lonza), used as positive control of matrigel-invasion. Cells were incubated in a humidified incubator at 37°C with 5% CO2 for 48 h. Afterwards, cells were fixed in 4% paraformaldehyde (Sigma) for 2 min at room temperature, permeabilized with 100% of Methanol (Sigma) for 20 min at room temperature, and stained with crystal violet (Sigma). The non-invading cells that remained on the upper surface of the membrane were removed by scraping while the invaded cells were counted using an inverted microscope at a magnification 400X in at least 8 randomly selected fields using IAS 2000 System (Delta Sistemi, Rome, Italy).

### Real-time PCR

A constant amount of RNA (0,5 μg/sample) was retro-transcribed into complementary DNA (cDNA) and then 1 μl of cDNA/sample was amplified using the following conditions: denaturation 1 minute at 95°C; annealing 30 seconds, at 60°C for β-Actin and IL-34 followed by 30 seconds of extension at 72°C. Primer sequences: IL-34: forward, 5′- ACAGGAGCCGACTTCAGTAC-3′ and reverse, 5′- ACCAAGACCCACAGATACCG-3′; β-actin: forward, 5′-AAGATGACCCAGATCATGTTTGAGACC-3′ and reverse 5′-AGCCAGTCCAGACGCAGGAT-3′. mRNA expression was calculated relative to the housekeeping β-Actin gene on the base of the Δ ΔCt algorithm.

### Total protein extraction and Western blotting

Paired tissue samples of tumoral and non-tumoral areas and colon cancer cells were lysed on ice in buffer containing 10 mM HEPES (pH 7.9), 10 mM KCl, 0.1 mM EDTA, 0.2 mM EGTA and 0.5% Nonidet P40 supplemented with 1 mM dithiothreitol, 10 mg/ml aprotinin, 10 mg/ml leupeptin, 1 mM phenylmethylsulfonyl fluoride, 1 mM Na3VO4 and 1 mM NaF. Lysates were clarified by centrifugation at 4°C, 12.000 × g for 30 minutes, and separated on 10% sodium dodecyl sulphate-polyacrylamide gel electrophoresis. IL-34, M-CSFR-1, PTP-z, phosphorylated (p) ERK1/2, ELK-1, p-p38, p-STAT3 and p-NF-kB p65 were detected using the following antibodies: mouse anti human IL-34 (1:1000 Abcam, Cambridge, UK), rabbit anti-human M-CSFR-1 (1:500 Novus Biological, Littleton, CO, USA), rabbit anti-human PTP-z (1:1000 Abcam), mouse anti-human p-ERK 1/2 (1:500 Santa Cruz Biotechnology, Inc., Dallas, TX, USA), mouse anti-human p-ELK 1 (1:500 Santa Cruz Biotechnology, Inc., Dallas, TX, USA), rabbit anti-human p-p38 (1:1000 EMD Millipore Corporation), rabbit anti human p-STAT3 Tyr705 (Cell Signaling, Danvers, MA, USA), rabbit anti human p-NF-κB/p65 Ser536 (Cell Signaling), respectively, followed by horseradish peroxidase–conjugated secondary IgG monoclonal antibodies (all used at 1:20000 final dilution, Dako, Milan, Italy). The reaction was detected with a sensitive enhanced chemiluminescence kit (Pierce, Rockford, IL, USA). After the analysis, blots were stripped and incubated with the following internal loading controls: mouse anti-human β-actin antibody (final dilution 1:5000 Sigma-Aldrich), rabbit anti-human total ERK1/2 (1:500, Santa Cruz Biotechnology), mouse anti-human total p38, rabbit anti- human NF-κB/p65 and mouse anti-human total STAT3 (at 1:500, Santa Cruz Biotechnology) followed by horseradish peroxidase–conjugated secondary IgG monoclonal antibodies (all used at 1:20000 final dilution, Dako).

### Immunohistochemistry

Immunohistochemistry was performed on archival frozen sections of paired tumoral and non-tumoral samples of 8 CRC patients. Freshly obtained samples were embedded in a cryostat mounting medium (Neg–50 Frozen Section Medium, Thermo Scientific, Langenselbold, Germany), snap frozen and stored at –80°C. Six μm-thickened sections were mounted onto superfrost plus glass slides (Thermo Scientific) and fixed in 4% neutral buffered formalin for 10 minutes at room temperature and then in increasing ethanol solutions. After washing in Tris Buffered Saline, endogenous peroxidase activity was quenched with 3% H_2_0_2_ diluted in methanol for 10 minutes at room temperature. The slides were incubated with a mouse monoclonal antibody directed against human IL-34 (final dilution 1:50000, Abcam, Cambridge, UK) at room temperature for 1 hour followed by a biotin-free HRP-polymer detection technology (Ultravision Detection System, Thermo Scientific, Waltham, MA, USA) with 3,3′diaminobenzidine (DAB) as a chromogen (Dako). The sections were counterstained with haematoxylin, dehydrated and mounted. Isotype control IgG-stained sections were prepared under identical immunohistochemical conditions as described above, replacing the primary antibody with a purified mouse normal IgG control antibody (R&D Systems). The IL-34-positive cells were counted in at least 6 fields per section using IAS 2000 System (Delta Sistemi, Rome, Italy) and expressed as number of cells for high power field (hpf). Moreover, immunohistochemistry was performed on formalin-fixed paraffin-embedded sections of non-tumoral and tumoral samples of 6 CRC patients. The sections were deparaffinized and dehydrated through xylene and ethanol and the antigen retrieval was performed in Tris EDTA citrate buffer (pH 7.8) for 30 minutes in thermostatic bath at 98°C (Dako). Immunohistochemical staining was performed using a rabbit monoclonal antibody directed against human M-CSFR-1 (final concentration 1:200, Novus Biological) or rabbit anti-human PTP-z (1:100 Abcam) at room temperature for 1 hour followed by a biotin-free HRP-polymer detection technology with DAB as a chromogen (MACH 4 Universal HRP-Polymer Kit, Biocare Medical). The sections were counterstained with haematoxylin, dehydrated and mounted. Isotype control IgG-stained sections were prepared under identical immunohistochemical conditions as described above, replacing the primary antibody with a purified mouse or rabbit normal IgG control antibody.

### Enzyme-linked immunosorbent assay (ELISA)

Human IL-34 was measured using a sensitive commercial ELISA kit (R&D Systems) according to the Manufacturer’s instructions.

### Statistical analysis

Differences between groups were compared using the Student’s *t*-test and Wilcoxon test. All the analyses were performed using Graph-Pad 5 software.

## SUPPLEMENTARY MATERIALS FIGURES


