# Supplementary material for: Interleukin-34 sustains pro-tumorigenic signals in colon cancer tissue
Source: Oncotarget. 2017 Dec 15;9(3):3432–45. doi: 10.18632/oncotarget.23289 (PMC5790474; doi:10.18632/oncotarget.23289)
Supplement: Supplementary file 1 [file oncotarget-09-3432-s001.pdf]

Interleukin-34 sustains pro-tumorigenic signals in colon cancer tissue

SUPPLEMENTARY MATERIALS

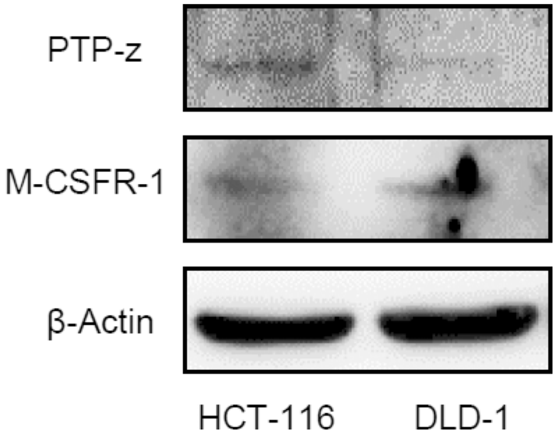

Supplementary Figure 1: Expression of Macrophage colony-stimulating factor -1 receptor (M-CSFR-1) and Receptor-type tyrosine-protein phosphatase zeta (PTP-z) in human cancer cell lines. Representative Western blots showing PTP-z, M-CSFR-1 and β-actin in HCT-116 and DLD-1.

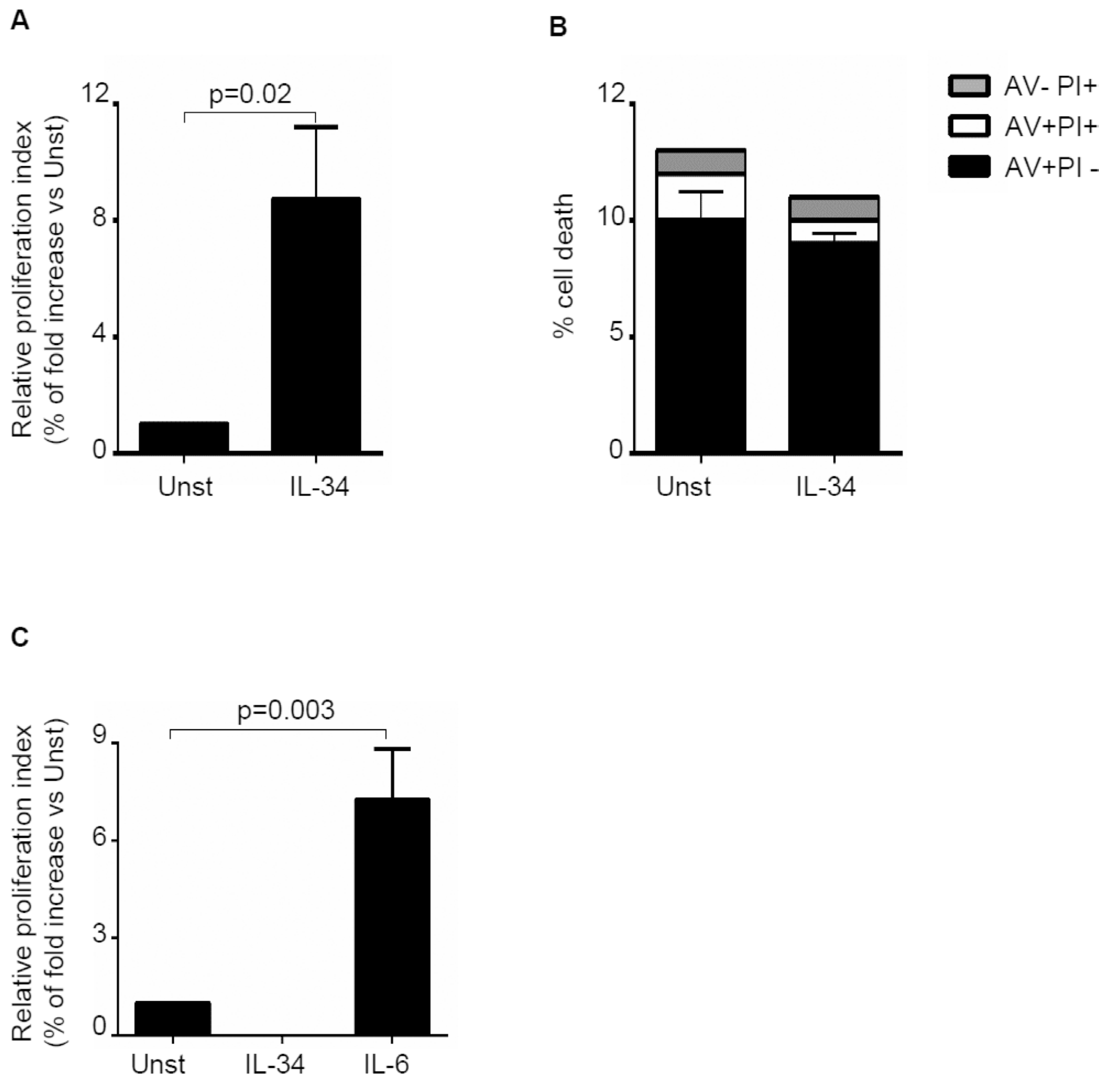

Supplementary Figure 2: Interleukin-34 (IL-34) induces CRC cell proliferation without affecting cell death. (**A**) Serum-starved HT-29 cells were either left unstimulated (Unst) or stimulated with recombinant human IL-34 (50 ng/ml) for 48 hours. Cell proliferation was evaluated by flow cytometry and proliferation index was calculated with Modfit LT. Data indicate mean ± SEM of 4 independent experiments. (**B**) Serum-starved HT-29 cells were either left unstimulated (Unst) or stimulated with recombinant human IL-34 (50 ng/ml) for 48 hours. Data indicate the percentage of cell death as assessed by flow cytometry analysis of Annexin V (AV) and/or Propidium Iodide (PI)-positive cells and are expressed as mean ± SEM of 4 experiments. (**C**) HCEC-1CT cells were either left unstimulated (Unst) or stimulated with recombinant human IL-34 (50 ng/ml) or recombinant human IL-6 (50 ng/ml) used as positive control for 72 hours. Cell proliferation was evaluated by flow cytometry and proliferation index was calculated with Modfit LT. Data indicate mean ± SEM of 4 independent experiments.
